# Evaluation of Commonly Used Algorithms for Thyroid Ultrasound Images Segmentation and Improvement Using Machine Learning Approaches

**DOI:** 10.1155/2018/8087624

**Published:** 2018-09-23

**Authors:** Prabal Poudel, Alfredo Illanes, Debdoot Sheet, Michael Friebe

**Affiliations:** ^1^Department of Medical Engineering, Otto-von-Guericke University Magdeburg, 39106 Magdeburg, Germany; ^2^Department of Mechanical Engineering, Indian Institute of Technology Kharagpur, 822113 Kharagpur, India

## Abstract

The thyroid is one of the largest endocrine glands in the human body, which is involved in several body mechanisms like controlling protein synthesis and the body's sensitivity to other hormones and use of energy sources. Hence, it is of prime importance to track the shape and size of thyroid over time in order to evaluate its state. Thyroid segmentation and volume computation are important tools that can be used for thyroid state tracking assessment. Most of the proposed approaches are not automatic and require long time to correctly segment the thyroid. In this work, we compare three different nonautomatic segmentation algorithms (i.e., active contours without edges, graph cut, and pixel-based classifier) in freehand three-dimensional ultrasound imaging in terms of accuracy, robustness, ease of use, level of human interaction required, and computation time. We figured out that these methods lack automation and machine intelligence and are not highly accurate. Hence, we implemented two machine learning approaches (i.e., random forest and convolutional neural network) to improve the accuracy of segmentation as well as provide automation. This comparative study intends to discuss and analyse the advantages and disadvantages of different algorithms. In the last step, the volume of the thyroid is computed using the segmentation results, and the performance analysis of all the algorithms is carried out by comparing the segmentation results with the ground truth.

## 1. Introduction

The segmentation and volume computation of thyroid are of prime importance when it comes to the diagnosis and treatment of thyroid diseases. Thyroid is a butterfly-shaped gland located below the Adam's apple on the front of the neck. Most of the thyroid diseases like Graves' (excessive production of thyroid hormones), subacute thyroiditis (inflammation of thyroid), thyroid cancer, goitre (thyroid swelling), and thyroid nodule (small abnormal lump growths in thyroid) involve changes in the shape and size of thyroid [1]. Hence, it is essential to compute the volume of thyroid over time to identify whether the thyroid is healthy or not. We use ultrasound (US) imaging for data acquisition instead of other imaging modalities as it is much safer and painless when used on the patients.

Several research works have been proposed on how to segment the thyroid in individual 2D US images. Zhao et al. [2] proposed several approaches (edge detection, method of threshold value, region splitting and merging, watershed segmentation, active contour, graph theory, US image segmentation based on Ncut, and segmentation based on improved normalized cut) based on 2D segmentation of thyroid in US images. Kaur and Jindal [3] segmented thyroid from 2D US and scintigraphy images using active contour without edges, localized region-based active contour, and distance regularized level set. Augustin et al. [4] tested and segmented thyroid US images using fuzzy c-means algorithm, histogram clustering, QUAD tree, region growing, and random walk [5]. A polynomial Support Vector Machine (SVM) was used [6] to segment the thyroid gland in US images. A local region-based active contour was proposed [7] to segment and compute the area of segmented thyroid in a 2D US image. Another region-based active-contour implementation to segment medical images was carried out by Mylona et al. [8, 9] where they encoded the local geometry information (i.e., orientation coherence in the edges of the regions to be segmented) to control the evolution of the contour. Similarly, thyroid segmentation in US images using a novel boundary detection method and local binary patterns for texture analysis was proposed by Keramidas et al. [10]. Level-set active-contours models for thyroid segmentation in US images were used in [11, 12]. These methods were mainly based on variable background active contour and joint echogenicity texture. Garg and Jindal later worked on feedforward neural network [13] to segment thyroid gland from US images. Recently, Narayan et al. [14] made use of the speckle-related pixels and imaging artefacts as source of information to perform multi-organ (thyroid, carotid artery, muscles, and trachea) segmentation in thyroid US images.

Similarly, several research works have been carried out to segment a full 3D thyroid image. Kollorz et al. [15] proposed a semi-automated approach to classify thyroid gland for volumetric quantification using geodesic active contour. Chang et al. [16] proposed radial basis function (RBF) neural network to segment the blocks of thyroid gland. 3D mass spring models for thyroid cartilage segmentation by creating a 3D deformable shape models were proposed by Dornheim et al. [17] but on computed tomography (CT) images. A complete segmentation and analysis of 3D thyroid images was carried out by Osman [18] by thresholding the voxel intensities and then connecting similar voxels to predict the segmenting regions.

The aforementioned approaches have limitations in the sense that they work either on a single 2D image or on a whole 3D image and they do not make use of the spatial relationship between the neighbouring US slices. Hence, we propose three widely used segmentation algorithms which usually work on a 2D image but can be extended to segment a sequence of freehand US images by making use of the spatial relationships between the corresponding image frames. These three approaches are based on active contours without edges (ACWE), graph cut (GC), and pixel-based classifier (PBC). In case of ACWE, the centroid of the segmented image is used as the priori information to find the location of contour initialization in the corresponding slices. GC allows the user to select the foreground and background areas in one image, and these information are transferred to the corresponding slices for further initialization. In PBC, the user clicks inside and outside of the thyroid region to extract the features for thyroid segmentation which are used to train the decision trees and later to classify thyroid and nonthyroid regions in the corresponding images. Our approach of segmenting individual slices and then reconstructing them to a volume possess greater advantages than segmenting directly on the volume itself as segmenting on the 3D image is very complex and is difficult to control. Also, segmentation in 2D allows to analyse the shape of the thyroid in detail as compared to segmenting directly on the thyroid volume.

The main purpose of this work is to compare three nonautomatic segmentation techniques, which are based on ACWE [19], GC [20], and PBC [16] to perform the segmentation in the thyroid images. They are compared based on their accuracy, robustness, ease of use, and computation time. We also compare the results of these approaches to some of the existing methods [17] that use mass spring models. These algorithms were chosen over others as they can be used not only on one image but also on a sequence of US images in a dataset to produce a 3D segmented thyroid as the information from a segmented image could be transferred to the corresponding image slices to segment them. Additionally, when the segmentation is ongoing in different images in a dataset, the user could directly interact with the segmentation results and correct them if there are any under- or oversegmentation. After segmentation, the segmented images are later used for 3D reconstruction and volume computation using ImFusion [21] and MeVisLab [22]. We figured out that the nonautomatic methods pose several disadvantages and thus implemented two automatic machine learning based methods such as Random Forest Classifier (RFC) and Convolutional Neural Network (CNN) and compared their performance with the nonautomatic methods. We came to the conclusion that the commonly used algorithms could not segment a series of US images highly accurately as compared with these supervised learning techniques.

## 2. Materials and Methods

In this section, we will explain the three nonautomatic as well as the two automatic methods that are compared in this work to segment the thyroid glands in US images. The automatic methods use 3D thyroid images while the nonautomatic methods use 2D images. We will also present the 3D reconstruction (using segmented results from nonautomatic methods) as well as volume computation technique.

### 2.1. Active Contour without Edges

#### 2.1.1. Preprocessing

ACWE segmentation was followed by a preprocessing step as the algorithm mainly worked on the gradient information for contour evolution and the preprocessing step improved the gradient visualization. US images mainly contain speckle noise [23] and have low contrast [16]. The speckle noise is produced by the interference of the returning ultrasound waves at the transducer aperture as the ultrasound images are produced when the reflected sound waves from different surfaces inside the body are picked up by the transducer. To enhance the contrast and suppress the speckle noise, a preprocessing step is carried out. Contrast enhancement [24] is used to improve the visibility of the thyroid region. In this work, we make use of Histogram equalization technique which is one of the methods used in contrast enhancement. It helps in recovering the lost contrast in the image by remapping the brightness values such that they are distributed over all the pixels. After histogram equalization, median filter is applied to suppress the speckle noise. It not only reduces speckles but also preserves the gradient/edge information.

#### 2.1.2. Segmentation

After preprocessing, the segmentation process was carried out using the level-set approach developed by Chan and Vese [19]. It is based on the minimization of the Mumford-Shah functional and involves four main steps.

**Figure 1:**
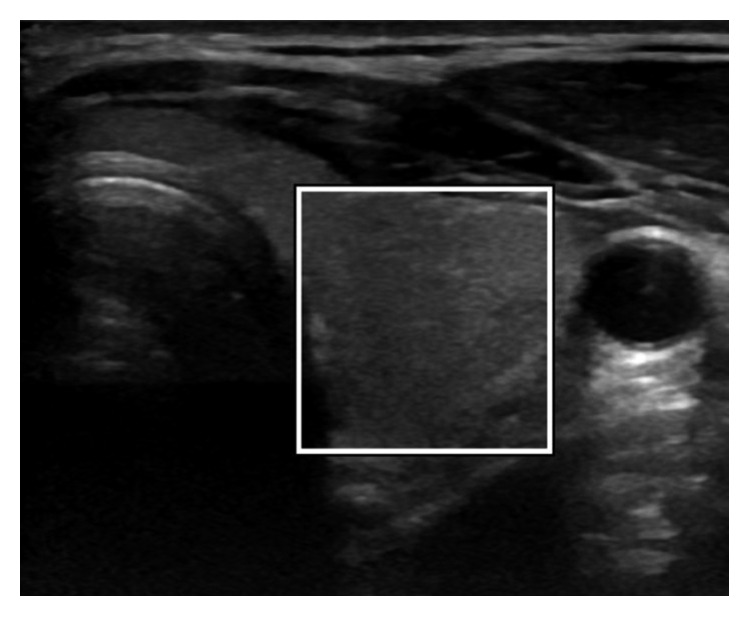
ACWE initialization of the mask by the user.

In the first step, the user starts by initializing a rectangle/square mask around the region to be segmented. The initialization of the mask (Figure 1) is a very important step in this algorithm as a wrong initialization can lead to the segmentation of unnecessary segments inside the image. The initial mask separates the image into two regions: foreground (=1) which is inside of the mask and the background (=0) which is outside of the mask.

In the second stage, a Signed Distance Function (SDF), ∅, is computed from the initial mask (*C*) by using the Euclidean distance. SDF is one of the methods of representing the level sets which are used to keep track of evolving curve over time. Our goal is to evolve ∅(*x*, *y*) when the evolving contour (*C*) is the zero level set of ∅(*x*, *y*, *t*) at each time *t*.

Thirdly, the forces that control the evolution of the initial contour are computed. These forces are force from the image and force from curvature. Hence, they are calculated as follows:

(1) $$\begin{array}{c} F_{\text{image}}=\underset{\text{inside}\;\;C}{\int }{\left(I-{µ}_{\text{in}}\right)}^{2}+\underset{\text{outside}\;\;C}{\int }{\left(I-{µ}_{\text{out}}\right)}^{2}, \end{array}$$

where *I* is the image, µ_in_ is the average inside the contour, and µ_out_ is the average outside the contour.

(2) $$\begin{array}{c} F_{\text{curvature}}=\frac{\emptyset_{x}^{2}*\emptyset_{yy}+\emptyset_{y}^{2}*\emptyset_{xx}-2\emptyset_{x}\emptyset_{y}\emptyset_{xy}}{{\left(\emptyset_{x}^{2}+\emptyset_{y}^{2}\right)}^{3/2}}. \end{array}$$

All the derivatives are computed using central differentiation method. Using these two forces, the equation of the curve is computed using the Taylor expansion given by the following equation:

(3) $$\begin{array}{c} \emptyset \left(\left(x,y\right),t+\Delta t\right)=\Delta t*\emptyset_{t}+\emptyset \left(\left(x,y\right),t\right), \end{array}$$

where

(4) $$\begin{array}{c} \emptyset_{t}=\alpha \;*\;F_{\text{curvature}}+\frac{F_{\text{image}}}{\max\left|F_{\text{image}}\right|}, \end{array}$$

(5) $$\begin{array}{c} \Delta t=\frac{1}{\max\left(\emptyset_{t}\right)+\varepsilon }, \end{array}$$

where *α* represents the smoothing term and *ε* represents the coefficient to satisfy Courant, Friedrichs, and Lewy (CFL) condition [25]. The evolution of the contour stops after the given number of iterations are complete, giving us the segmented thyroid image.

In the last stage, the result of the segmentation on the first image of the dataset is used to segment rest of the images in the dataset. After the segmented thyroid is obtained, its centre of mass is computed. This centre of mass is used to find probable centre of mass of the thyroid in next image slice.

It is computed by making use of the tracking matrices obtained during the data acquisition phase. Each image in the dataset has an associated tracking matrix which gives the transformation from the origin of electromagnetic (EM) tracking system to the centre of the image. Hence, the centre of each image can be computed using the transformation matrix which has the information about the centre of each image in the dataset.

**Figure 2:**
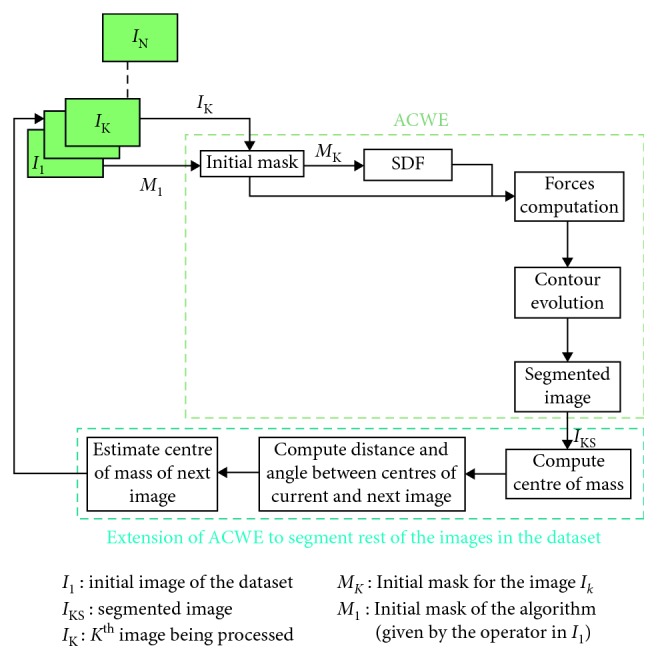
Schematic description of ACWE segmentation method.

Using this information, the Euclidean distance between the image centres of the current and the next image is computed and the angle between the centres is computed. After computing the distance and angle between the two image centres, a probable centre of mass of the thyroid in the next image is computed by traversing the same distance and angle from the centre of mass of the current segmented thyroid [26]. Centre of mass computed this way will serve to be the centre of rectangle in the next image frame around which the new mask will be initialized automatically. The size of the rectangle will be the same as it was drawn by the user in the first image. In this way, the automatic initialization of segmentation mask is done in the consecutive image frames which will undergo the ACWE algorithm to produce segmented thyroids. The schematic description of the approach is presented in Figure 2.

A fixed number of iterations is set by the user for the contour evolution. By increasing the number of iterations, the computation time will be higher. Hence, a trade-off between the accuracy and computation time has to be maintained while running this algorithm.

### 2.2. Graph Cut

**Figure 3:**
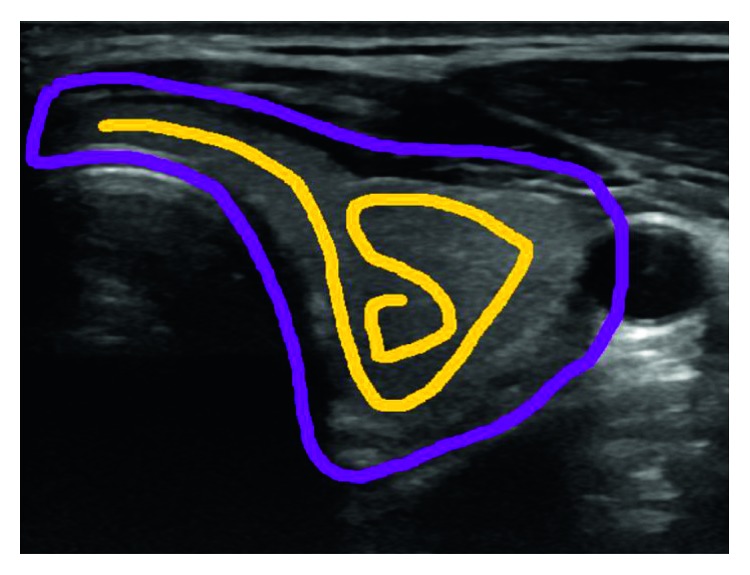
Graph cut initialization by the user.

**Figure 4:**
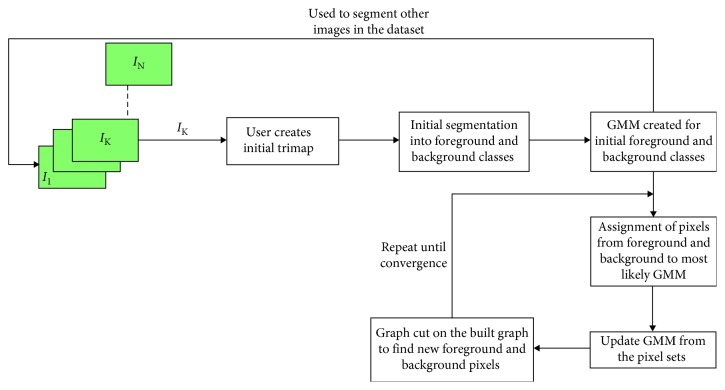
Schematic description of GC segmentation method.

This approach makes use of the GrabCut algorithm from Rother et al. [20]. It is also a semi-automatic 2D segmentation algorithm just like the ACWE as the user needs to mark the regions as being thyroid and nonthyroid in the initialization phase. It starts with the user creating an initial trimap by marking the thyroid region to be segmented by using yellow scribbles and the surrounding (i.e., nonthyroid) regions by using violet scribbles as seen in Figure 3. The pixels outside of the violet scribble are marked as known background, pixels inside of the violet scribble are marked as unknown, and the yellow scribble areas are marked as definite foreground. The schematic description of the approach can be seen in Figure 4.

Then, an initial image segmentation is computed where all the unknown pixels are placed in the foreground class and all the known background pixels are placed in the background class. These initial foreground and background classes are used to construct foreground and background Gaussian Mixture Models (GMMs) using the Orchard-Bouman clustering algorithm [27]. Each pixel in the foreground class is assigned to the most likely Gaussian component in the foreground GMM, and similarly, each pixel in the background class is assigned to the most likely background Gaussian component. With the new distribution of the pixels, the initial GMMs are disregarded and new GMMs are learned from the pixel distributions in each of the two classes.

Finally, a graph is built which consists of each pixel as node and two special nodes (i.e., foreground and background). All of these nodes are connected by two types of edges (also called as links). The first link (i.e., *N*-link) connects a pixel to its 8-neighbourhood pixels. These links describe the penalty for placing a segmentation boundary between the neighbouring pixels. The second link (i.e., *T*-link) connects each pixel to the foreground and background nodes. Each of these links has a weight which represents the probability of a pixel belonging to either a foreground or a background. These probabilities are computed in the GMM models and updated in each iteration until a convergence is reached to get a segmented thyroid. The weight of the *N*-links between pixel *m* and its 8-neighbourhood pixels, *n*, is computed as

(6) $$\begin{array}{c} N\left(m,n\right)=\frac{\gamma }{\text{dist}\left(m,n\right)}e^{-\beta {\left\|z_{m-z_{n}}\right\|}^{2}}, \end{array}$$

where *z*_*m*_ is the color of pixel *m*, *γ* = 50 as suggested by Blake et al. [28], and *β* is given as follows by Boykov and Jolly [29]:

(7) $$\begin{array}{c} \beta =\frac{1}{2\langle {\left\|z_{m}-z_{n}\right\|}^{2}\rangle }. \end{array}$$

The initial user initializations are interpolated in the corresponding slices to mark the thyroid and nonthyroid regions (i.e., foreground and background) and create corresponding GMMs. The aforementioned processes are then repeated in each individual images to segment all the thyroid in the dataset. The advantage of this algorithm over ACWE is that it is much faster than ACWE and the user can interact with the result of the segmentation (i.e., postsegmentation) and correct if any errors are present. The results of the segmentation from GC in all the 2D images are used to reconstruct the 3D thyroid by using MeVisLab [22]. The 3D model is updated as soon as the user tries to improve the segmentation results by further interaction in the segmented images. Thus, the accuracy of the algorithm is directly proportional to the number of user interactions on the segmented images.

The increased number of user interactions adds to the computation time of the algorithm. Hence, an optimum number of user interaction should be chosen to obtain the best segmentation results with minimum user interaction. For this purpose, the user interaction in every 10 slices or every 2 mm was proposed.

### 2.3. Pixel-Based Classifier

This approach is based on training the decision trees by using different features computed from the images. In this work, three image features are computed. The selection of the features is based on the work of Chang et al. [16]. The first feature that is computed is the coefficient of variation *C*_*v*=(*σ*/*µ*)_, where σ means the standard deviation and *µ* is the mean of the user selected region during the initialization process. This coefficient is computed in two different sized neighbourhoods (i.e., 4-neighbours and 8-neighbours) of every pixel, thus resulting in two features. The third feature that is computed is the mean of the smaller of the two neighbourhoods. So, the first two features are the coefficient of variation at two different sized neighbourhoods of every pixel, and the third feature is the mean of the smaller of the two neighbourhoods.

**Figure 5:**
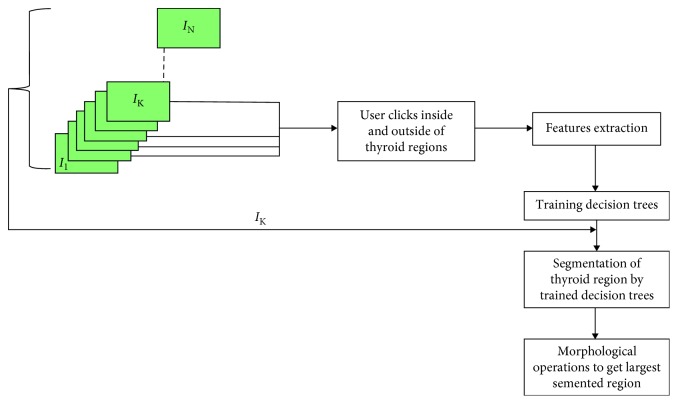
Schematic description of PBC segmentation method.

The algorithm starts by the user clicking on the inside and outside of the thyroid in several thyroid images from where the features are computed which are then passed as training input for the decision trees. The trained trees later classify the different regions in the image as thyroid or nonthyroid. After segmentation, the user can click in more regions to improve the segmentation results. However, selection of wrong thyroid regions for training the decision trees might result in oversegmentation. So, the user should carefully select the thyroid regions. The presented approach is shown in the schematic diagram in Figure 5.

After segmentation and correction of the segmentation errors, still some regions which are not part of thyroid might exist. In order to eliminate these regions, morphological operations were carried out to find the largest connected component which is then considered to be the final segmented thyroid.

We also tried to test this approach by using additional features as presented in Chang et al., but we found out that these features only slightly improved the segmentation accuracy, while the computation time increased significantly. Hence, we selected only the three aforementioned features and trained our decision trees with them. Similarly, we chose to work with decision trees instead of radial basis function (RBF) neural network as used in Chang et al. because of its faster computation time.

This approach is the most intuitive one and requires the least user interaction. However, the user can select more than one thyroid region during the initialization phase, and the features are computed accordingly. So, the user should wisely select the regions that are only the part of thyroid.

### 2.4. Random Forest Classifier

This approach is based on training a random forest classifier for a binary classification problem, which classifies each of the voxel in the thyroid US images as thyroid or nonthyroid. RFC is basically a type of ensemble learning method which constructs a final classifier using a set of *M* individual weak classifiers. In our case, we created 12 binary decision trees of depth 10.

We trained our RFC using a 10-fold cross validation technique where 9 datasets were used for the training and 1 as validation data for testing the trained model. This was repeated until all the 10 datasets were used for testing at different iterations. The RFC approach uses some typical out of the box image processing features including gradients, Laplacian, Gaussian blur, and resampling at various resolutions, making it a total of 30 different features for training the decision trees. These features are computed in each voxel with voxel size 15.

The input from the training data for each of the trees, *x* ∈ {1,…, *M*}, in the ensemble is created by using bootstrapping of the samples (bagging) from the training dataset and randomly sampling the subset of the features supplied to each tree. Each tree is a collection of nodes *N* and features *F*, which aid to final classification result. A decision tree is made up of a single parent node *N*_*p*,*x*_, multiple splitting nodes *N*_*s*,*x*,*i*_, ∀*i* ∈ {1,…*k*}, and leaf nodes *N*_*l*,*x*,*j*_, ∀*j* ∈ {1,…*p*}. During splitting of the nodes, the best split is not chosen based on all the features but a random subset of features from the training dataset.

All the leaf nodes inside a decision tree will have a final probabilistic model ∅_*x*,*j*_ ∈ [0,1] associated with it. The final decision of a forest for each of the patches extracted from the US images is made by averaging the individual decisions (∅_*x*,*j*_(*p*)) from all the individual trees in the forest.

(8) $$\begin{array}{c} P^{\text{RF}}\left(y\left(p\right)=1\right)=\frac{1}{M}\overset{M}{\underset{x=1}{\sum }}\emptyset_{x}\left(p\right). \end{array}$$

We have used the most common and recognized method to train the classifier [30, 31]. The implementation of RFC is carried out using ImFusion [21].

### 2.5. Convolutional Neural Network

**Figure 6:**
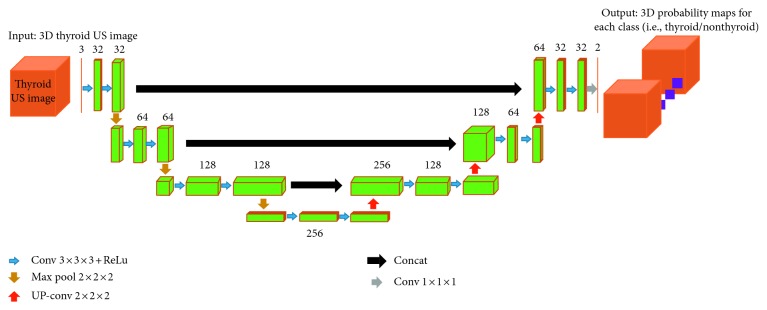
Architecture of our 3D U-net CNN. Each green box represents the feature maps.

This approach is based on training of the CNN using the U-net architecture (Figure 6) proposed by Ronneberger et al. [32] which consists of encoder and decoder parts that analyse the whole image by contracting in each successive layers and then expanding in order to produce a full-resolution segmentation, respectively. Just like RFC, the training and testing of CNN is performed using a 10-fold cross validation technique. The input for the CNN consists of a 3D thyroid US images and its corresponding ground truth. The input can be represented as *D*=(*I*_*n*_, *G*_*n*_), where *I*_*n*_ denotes one of the thyroid US image and *G*_*n*_ denotes its ground truth obtained from medical experts.

The network consists of two paths (i.e., downsampling/encoder/left side and upsampling/decoder/right side). The downsampling path consists of two 3 × 3 × 3 convolutions followed by a rectified linear unit (ReLu) in each layer and then a 2 × 2 × 2 max pooling with stride of 2 in each dimension. The number of feature channels is doubled in each downsampling step. The upsampling path remaps the lower resolution feature maps to the higher resolution space of the input images. It does this by upsampling the feature maps followed by a 2 × 2 × 2 convolution (upconvolution) which halves the number of feature channels in each upsampling step, a concatenation with the corresponding feature map from the downsampling path and two 3 × 3 × 3 convolutions, each followed by a ReLu activation. The final convolution layer uses a 1 × 1 × 1 convolution with a voxel-wise softmax activation function to compute a 3D probability map for each of the target label (i.e., thyroid or nonthyroid) as the output of our network.

Since the available datasets were only with 10 datasets consisting of 1416 images, we had to make sure that the network was not overfitting. We performed data augmentation by rotating the images at random angles between −10° and +10°, translating between −20 and +20 voxels in each dimension, and scaling between −1.5 and 1.5 times from the original size, and since the thyroid are in the left and right sides in the human body, we also flipped the images. We added a dropout of 25% after each pooling layer so that the unnecessary neurons are discarded. Finally, we used Adam optimizer with relatively low learning rate to make sure that the network was not overfitting. During the training, we observed that the validation accuracy was very close to that of the training accuracy which proves that our network was not overfitting.

#### 2.5.1. 3D Reconstruction and Volume Computation

This step involves 3D reconstruction and volume computation of the segmented thyroid from ACWE and PBC using ImFusion [21] and GC using MeVisLab imaging tools. The segmented 2D images are stored as binary images which are processed to make a video. The video file is passed along with the tracking data to ImFusion and MeVisLab for 3D reconstruction of the thyroid as well as volume computation. The reconstruction is done by the interpolation between the corresponding image frames of the ultrasound sweep to fill the empty spaces between the image slices.

Volume computation of thyroid is particularly important for the medical doctors as this allows them to keep track of the size of the thyroid over time and diagnose whether the patients have any thyroid disorders or not.

## 3. Experimental Results

### 3.1. Data Collection

We acquired the thyroid datasets from different clinical university hospital-based sources. A total of 6 healthy human datasets were acquired using the General Electric (GE) Logiq E9 US system which was equipped with the Ascension driveBay EM tracking system. These dataset along with the ground truth are available at Open-CAS [33]. A ML6-15 linear probe was used to acquire the data. All the images were acquired along with a tracking matrix that gave the transformation from the origin of the EM tracking system to the centre of the image. These matrices are used for the 3D reconstruction of the segmented thyroid. The images for the evaluation of nonautomatic methods had a size of 760 × 500 pixels.

A total of 1416 2D images corresponding to 10 datasets were acquired and used for the evaluation of both the automatic and nonautomatic methods. The 3D models of all the 10 datasets were used for evaluating the automatic methods. All these datasets are stored in the DICOM format. To evaluate the accuracy of our segmentation approaches, we acquired the ground truth by manually tracing the thyroid contour with the help of two medical experts from Magdeburg university clinic using MeVisLab. The datasets are presented in Table 1. The results and discussion may be presented separately, or in one combined section, and may optionally be divided into headed subsections.

### 3.2. Evaluation Procedure

For evaluation of the segmented images, we compare all the segmentation algorithms using two performance measures. We compute Dice's coefficient (DC) to compare the segmentation accuracy between active contours, graph cut, and pixel-based classifier. Similarly, we compute Hausdorff distance (HD) to compare the accuracy of all the algorithms with the works of Dornheim et al. [17]. These measures are computed by comparing the segmentation results with the ground truth images.

*Dice's coefficient* is a numerical estimate used for comparing the similarity of two samples. In our case, it is a measure to see how accurate our segmented results were by comparing the segmentation results with the ground truth obtained by manual segmentation of the thyroid by trained medical staff. It ranges from 0 to 1, 0 meaning the two datasets are completely different from each other and 1 meaning the datasets completely overlap with each other. It is computed by using the following formula:

(9) $$\begin{array}{c} \text{Dice's}\;\;\mathrm{coefficient}=\frac{2\left|X\;\cap \;Y\right|}{\left|X\right|+\left|Y\right|}, \end{array}$$

where *X* is the segmented image and *Y* is the ground truth.

Similarly, Hausdorff distance measures how far the two subsets of a metric space are from each other. In other words, it is the greatest of all the distances from a point in one set to the closest point in the other set; so the less the distance is, the closer the sets are. It is computed by using the following formula:

(10) $$\begin{array}{c} \text{Hausdorff}\;\;\mathrm{distance}=\underset{x\in X}{\max}\left\{\underset{y\in Y}{\min}\left\{d\left(X,Y\right)\right\}\right\}, \end{array}$$

where *x* are the pixels in the segmented image *X* and *y* are the pixels in the ground truth image *Y*.

The results of segmentation are later used for 3D reconstruction and volume computation. We compare the volumes of the segmented thyroid obtained from all the five algorithms. The accuracy of volume computation is computed by comparing the volume of the 3D reconstructed segmented thyroid to that of the ground truth.

### 3.3. Analysis of Segmentation and 3D Reconstruction

This section is further divided into two subsections where the first subsection will present the visual analysis of the segmented images and the second subsection will present the quantitative comparison of accuracy, robustness, ease of use, and computation time of all the segmentation algorithms that we have discussed.

#### 3.3.1. Visual Analysis

As mentioned earlier, a total of 1416 images in the ten datasets were taken for the evaluation procedure. An example of segmented thyroid US image from each of the proposed algorithms will be presented in this section.

**Figure 7:**
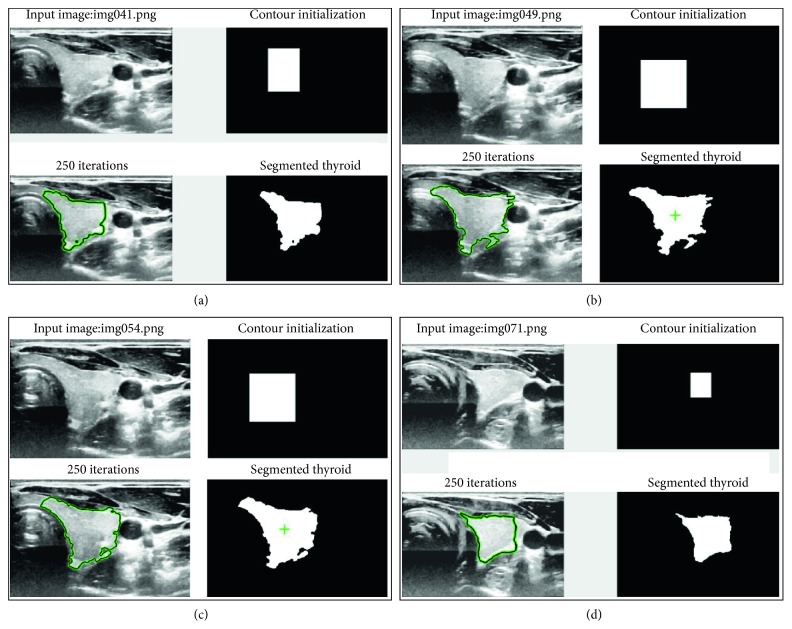
Segmentation of 4 different thyroid images using ACWE.

**Figure 8:**
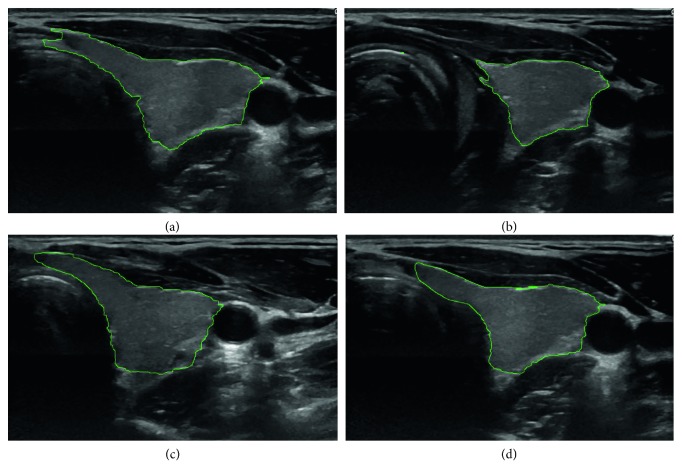
Segmentation of 4 different thyroid images using GC.

**Figure 9:**
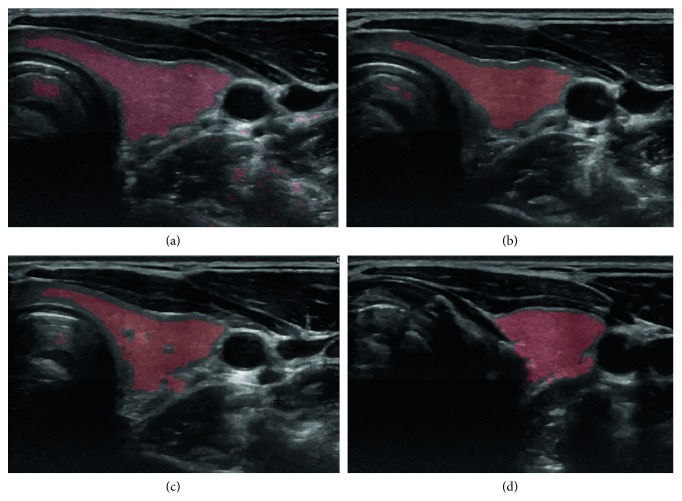
Segmentation of 4 different thyroid images using PBC.

**Figure 10:**
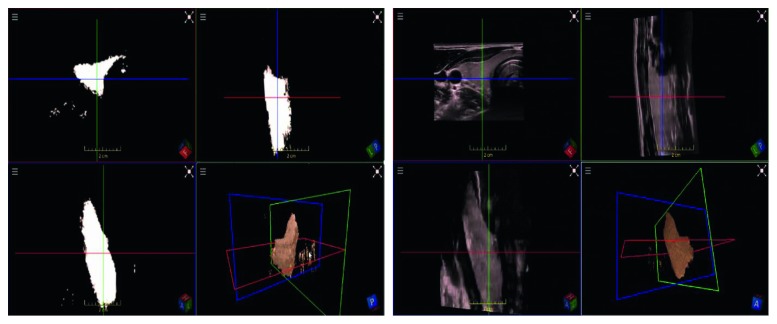
Segmentation of thyroid using RFC. Left: segmented thyroid images as binary images with three different viewing angles (top-left, top-right, and bottom-left, and 3D thyroid in bottom-right). Right: original thyroid images with three different viewing angles (top-left, top-right, and bottom-left, and segmented 3D thyroid in bottom right).

**Figure 11:**
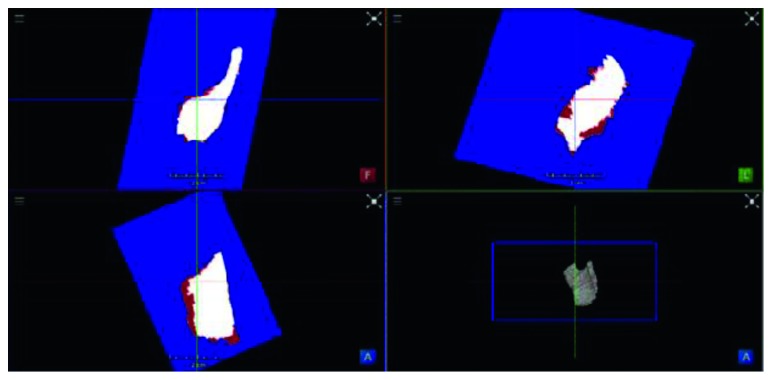
Comparison of the segmented thyroid (red) with the ground truth (white) using CNN. Top-left, top-right, and bottom-left: three different viewing angles of segmented thyroid and ground truth. Bottom-right: segmented thyroid in 3D.

The result of segmentation in four thyroid images using ACWE along with the user-initialized mask is shown in Figure 7, GC is shown in Figure 8, PBC is shown in Figure 9, RFC is shown in Figure 10, and CNN is shown in Figure 11. These segmentation results show that the automatic methods produce better segmented thyroid as compared to the nonautomatic methods. Within the nonautomatic methods, ACWE and GC give a better approximation of the segmented thyroid region compared to PBC as it has few oversegmented areas. In case of ACWE, the number of iterations of contour evolution is set by the user in order to optimize between the accuracy and the computation time, and because of this, the contour does not reach the narrow areas like the isthmus as shown in Figure 4. We also allow the user to stop the segmentation process where GC is more user-friendly as it allows the user to disregard the oversegmented areas in the postsegmentation stage. PBC works by computing the features from the areas the user select during the initialization process, and because of this, only those areas that have very similar features to that of initialized areas are segmented as thyroid region. This results in undersegmentation as well as oversegmentation most of the times.

**Figure 12:**
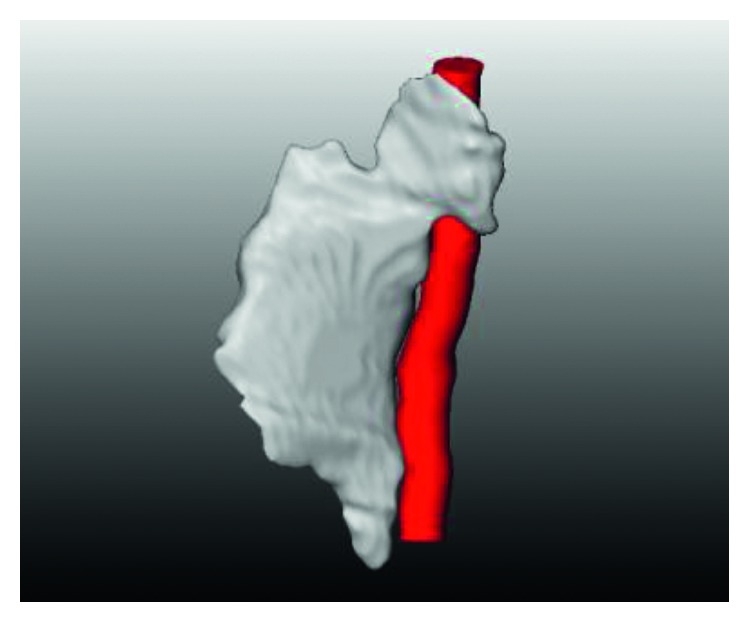
3D reconstructed thyroid using ImFusion.

**Figure 13:**
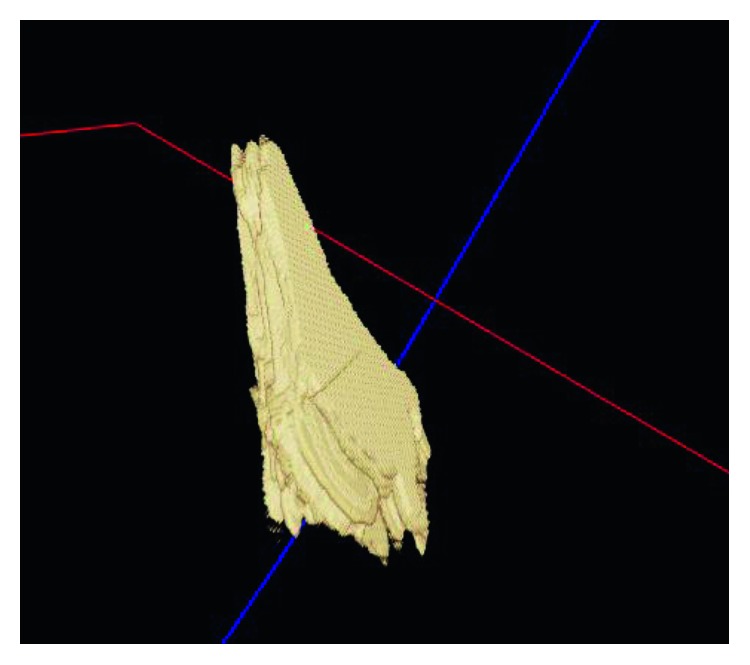
3D reconstructed thyroid (white) along with the neighbouring artery (red) using MeVisLab.

Similarly, for the visualization, we performed the 3D reconstruction of the segmented thyroid using the whole set of 2D segmented images. The 3D reconstructed thyroid using ImFusion is shown in Figure 12 and MeVisLab is shown in Figure 13. With MeVisLab, we could even segment the neighbouring artery (i.e., arteria carotis) using a Hessian-based vesselness filter [34].

#### 3.3.2. Quantitative Analysis

In this section, the comparison of the accuracy of segmentation in the all the five algorithms (i.e., ACWE, GC, PBC, RFC, and CNN) in terms of DC is presented in Table 2. Also, the comparison of segmentation accuracy of all the five algorithms with two of the standard algorithms [17] in terms of HD is presented in Table 3.

The volume of the segmented thyroids from ACWE, GC, and PBC was computed after the 3D reconstruction using ImFusion and MeVisLab and is presented in Table 4. The results of the volume computation from segmentation results show a close correlation with the segmentation results as well as ground truth in terms of accuracy.

We compared the three nonautomatic algorithms not only based on their accuracy of segmentation but also on other factors like the computation time, robustness of the algorithm, number of user interactions required, etc. All the algorithms performed differently on average where ACWE performed the best with an average DC of 0.800, PBC performed the worst with an average DC of 0.670, and GC performed relatively well with an average DC of 0.765. Even though ACWE was found to be the best performer, it is not accurate enough to use for clinical practices as they require relatively higher accuracy.

ACWE produced undersegmented and oversegmented results in some cases as the contour evolution (set by the user) does not reach all the regions of thyroid (e.g., isthmus of thyroid) as well as due to the wrong initialization of the contour (this happens when the segmentation results from one image frame are used to segment the corresponding image frames). In order to address these problems, the user could stop the ongoing segmentation at any image frame and change the number of iterations as well as re-initialize the initial mask. 7.7 re-initializations were required on average per dataset. Similarly, the average computation time for ACWE was around 369 seconds in average making it the slowest of all the algorithms, and the initialization determined the rest of the segmentation process. Hence, it is not very robust as compared to the other algorithms. All the methods were implemented in MATLAB in a Lenovo T430 ThinkPad notebook with Intel Core i5-3320M CPU, 2.60 GHz processor, and 8.00 GB RAM.

GC required the most number of user interactions (i.e., 36 scribbles on average) as the user could visualize the segmentation results instantly and improve it with more interactions. Hence, the quality of the results is directly proportional to the number of user interactions using this algorithm. The computation time was around 98 seconds on average per dataset. Graph cut is robust compared to the other two approaches as the user can control the results of the segmentation (i.e., during postsegmentation).

PBC required very few user interactions as the user had to click twice, one inside and one outside of the thyroid. However, the user could take more samples by additional clicks to improve the segmentation results. On average, 4.8 clicks were made while segmenting the images. In the same time, if wrong samples were taken, the user had to start the process from the beginning. This makes the algorithm less robust as compared to GC and ACWE. The computation time was around 10 seconds making it the fastest of all the algorithms. The comparison of the computation time and the number of user interactions required in all the three algorithms are shown in Table 5.

The RFC and CNN yielded an average DC of 0.862 and 0.876, respectively, in ten datasets when tested using a 9-fold cross validation. The computation time for the predication of one volume was on average 15.62 seconds for the RFC and 34.45 seconds for the CNN. These approaches had higher accuracies of segmentation as compared with ACWE, GC, and PBC. Also, these methods are highly robust as the algorithm does not depend on user interaction. Both of these approaches were also implemented in the same workstation as mentioned before.

## 4. Discussions and Conclusions

As mentioned earlier, it is essential to keep track of thyroid shape and size over time as it helps to diagnose whether the thyroid is healthy or pathological. In this paper, we have worked on three thyroid segmentation techniques which attempted to extend the 2D segmentation algorithm to generate a 3D segmented thyroid. We have evaluated these algorithms on the basis of accuracy of segmentation, computation time, number of user interactions required, and the robustness. At the same time, a comparison analysis was carried out with the works of Dornheim et al. [17].

We found that all three nonautomatic algorithms performed to different levels. However, a specific approach can be chosen if faster results are required or the least human interaction is desired. The result of volume computation corresponds to the segmentation as well as to the ground truth results which shows that the volume-rendering process was correct. The accuracy of the discussed algorithms could be further improved.

The computation time of ACWE could be accelerated by reducing the image resolution and using different initialization shapes (e.g., ellipse as thyroid is elliptical in shape). Similarly, the highly echogenic areas near the thyroid could be detected by preprocessing and later the evolution of the contour could be restricted to these areas which would reduce the oversegmentation. The preprocessing step can be further worked on with new contrast enhancement and filtering algorithms so that we generate a good quality ultrasound images before segmentation. In case of graph cut, a postprocessing step could be added which could take the shape prior information of the thyroid and remove the oversegmented areas automatically. Segmentation by pixel-based classifier could be improved with more image features. It can be made fully automatic using machine learning approaches and a postprocessing step to remove the oversegmented areas just like in graph cut. Also, advanced thresholding and connected component analysis could be performed to get the largest connected component and subsequently remove any elements outside that component to get a better segmented thyroid. Furthermore, all the acquired datasets were from healthy patients, so pathological datasets have to be acquired and tested on the discussed algorithms to evaluate their practical usefulness.

We figured out that the first three methods lacked automation and machine intelligence, were not highly accurate, and required long computation time. Hence, we implemented an RFC and a CNN that predict for each voxel the probability of belonging to the thyroid. Both approaches were trained for each voxel the probability of belonging to the thyroid in the available ten datasets, and they show better results as compared to the nonautomatic approaches.

As next steps, we will investigate several other thyroid segmentation approaches based on machine learning that operate directly on the volumetric three-dimensional ultrasound data instead of the 2D frames volumetrically compounded with isotropic spacing to form a 3D volume [35]. Similarly, future steps towards these automatic approaches must include more training data especially those with thyroid diseases as we have carried out our tests on healthy thyroid images only.

## Figures and Tables

**Table 1:** Acquired datasets for the evaluation of nonautomatic and automatic methods.

Dataset	Number of images
D1	96
D2	50
D3	94
D4	55
D5	135
D6	167
D7	216
D8	211
D9	201
D10	191
Total	1416

**Table 2:** Comparison of DC in five segmentation algorithms.

Dataset	ACWE	GC	PBC	RFC	CNN
D1	0.841	0.729	0.749	0.859	0.863
D2	0.819	0.636	0.666	0.864	0.876
D3	0.804	0.706	0.610	0.853	0.872
D4	0.816	0.841	0.680	0.872	0.869
D5	0.771	0.706	0.673	0.831	0.879
D6	0.781	0.853	0.623	0.853	0.874
D7	0.788	0.848	0.659	0.895	0.861
D8	0.746	0.746	0.547	0.877	0.888
D9	0.785	0.676	0.732	0.841	0.901
D10	0.852	0.912	0.761	0.875	0.877
Average	0.800	0.765	0.670	0.862	0.876

**Table 3:** Comparison of our approaches with other segmentation algorithms.

Dataset	Hausdorff distance (mm)
ACWE	8.1
GC	8.3
PBC	9.5
RFC	7.5
CNN	7.0
Volumetric mass spring model	11.1
Surface mass spring model	9.8

**Table 4:** Volume comparison of 2D segmented and 3D reconstructed thyroid to ground truth in cm^3^.

Dataset	ACWE	GC	PBC	Ground truth
D1	10.15	8.79	9.04	12.07
D2	11.46	8.90	9.30	13.99
D3	12.45	10.97	9.42	15.51
D4	11.91	13.82	9.86	14.64
D5	10.83	9.78	9.41	13.93
D6	12.18	13.95	10.90	9.86
D7	10.85	11.68	9.07	13.77
D8	10.66	10.66	7.82	14.29
D9	11.91	10.25	11.10	15.16
D10	10.52	11.26	9.40	12.35
Average	11.29	11.01	9.53	13.56

**Table 5:** Comparison of average computation time and number of interactions.

Approach	Computation time (sec)	Number of user interaction
ACWE	369	7.7
GC	98	36
PBC	10	4.8
RFC	15.62	None
CNN	34.45	None

## Data Availability

The data used for the evaluation purpose are uploaded in Open-CAS and available publicly (http://opencas.webarchiv.kit.edu/data/thyroid.zip).
